# Supplementary material for: Oxidative Inactivation of SARS-CoV-2 on Photoactive AgNPs@TiO2 Ceramic Tiles
Source: Int J Mol Sci. 2021 Aug 17;22(16):8836. doi: 10.3390/ijms22168836 (PMC8396237; doi:10.3390/ijms22168836)
Supplement: Supplementary file 1 [file ijms-22-08836-s001.zip › ijms-1345118-supplementary.pdf]

Oxidative Deactivation of SARS-CoV-2 on photoactive AgNPs@TiO_2 Ceramic Tiles

R. Djellabi[1*], N. Basilico[2], S. Delbue[2], S. D'Alessandro[3], S. Parapini[4], G. Cerrato[5], E. Laurenti[5], E. Falletta[1], C.L. Bianchi[1*]

[1]Department of Chemistry, University of Milan, , via Golgi 19, 20133, Milan, Italy
[2]Department of Biomedical, Surgical and Dental Sciences, University of Milan, Via della Commenda 10 20122 Milano, Italy. nicoletta.basilico@unimi.it (N.B.); serena.delbue@unimi.it (S.D.)
[3]Department of Department of Pharmacological and Biomolecular Sciences, University of Milan, Via Bal-zaretti 9, 20133 Milano , Italy . sarah.dalessandro@unimi.it
[4]Department of Biomedical Sciences for Health, University of Milan, Via Luigi Mangiagalli, 31, 20133 Milan, Italy
[5]Department of Chemistry, University of Turin, via P. Giuria 7 – 10125, Turin, Italy

***Corresponding authors:** claudia.bianchi@unimi.it; ridha.djellabi@unimi.it

Supplementary data

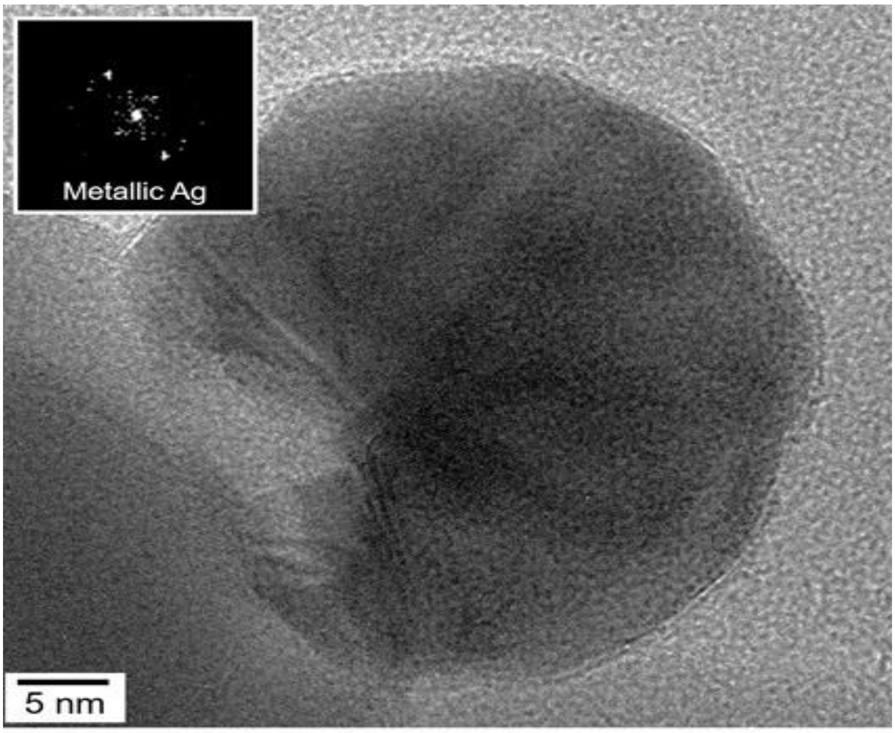

Figure S1. FFT analysis to check the form of Ag species in Ag@TiO_2 tile.

Table S1. Results of antiviral experiments on the surface of glass and Ag@TiO_2 tile in dark and light irradiation conditions.

**Surface**	**Light**	**Log PFU/cm^2 Time = 0 h**	**Log PFU/cm^2 Time = 4 h**	**Log PFU/cm^2 Time = 7 h**	**Log10 reduction**	**Viral inhibition %**
Glass	Dark	5.146	4.339		0.807	84.40
	LED	5.146	4.244		0.902	87.47
	UV	5.146	4.205		0.941	88.55
Ag-TiO_2 Tile	Dark	5.146	4.104		1.042	90.92
	LED	5.146	3.903		1.243	94.29
	UV	5.146	3.371		1.775	98.32
Glass	Dark	5.146		3.973	1.173	93.29
	LED	5.146		3.823	1.323	95.25
	UV	5.146		3.787	1.359	95.63
Ag-TiO_2 Tile	Dark	5.146		3.172	1.974	98.94
	LED	5.146		2.885	2.210	99.38
	UV	5.146		2.526	2.620	99.76

Table S2: Vero cells viability measured by MTT assay

	Adsorbance OD 650/550
Control	1.151 ± 0.151
Glass	1.293 ± 0.075
Ag@TiO_2 surface	1.209 ± 0.074

Data are the mean and standard deviation from three replicates.
